# DNA Damage Response and Cell Cycle Regulation in Pluripotent Stem Cells

**DOI:** 10.3390/genes12101548

**Published:** 2021-09-29

**Authors:** Andy Chun Hang Chen, Qian Peng, Sze Wan Fong, Kai Chuen Lee, William Shu Biu Yeung, Yin Lau Lee

**Affiliations:** 1 Department of Obstetrics and Gynaecology, Li Ka Shing Faculty of Medicine, The University of Hong Kong, 21 Sassoon Road, Hong Kong, China; andycch0@hku.hk (A.C.H.C.); szewan11@hku.hk (S.W.F.); bolkcad@gmail.com (K.C.L.); 2 Shenzhen Key Laboratory of Fertility Regulation, Reproductive Medicine Center, The University of Hong Kong Shenzhen Hospital, Shenzhen 518009, China; qianpeng_77@hotmail.com

**Keywords:** DNA damage, cell cycle regulation, pluripotent stem cells (PSCs), *FOXM1*, *SIRT1*

## Abstract

Pluripotent stem cells (PSCs) hold great promise in cell-based therapy because of their pluripotent property and the ability to proliferate indefinitely. Embryonic stem cells (ESCs) derived from inner cell mass (ICM) possess unique cell cycle control with shortened G1 phase. In addition, ESCs have high expression of homologous recombination (HR)-related proteins, which repair double-strand breaks (DSBs) through HR or the non-homologous end joining (NHEJ) pathway. On the other hand, the generation of induced pluripotent stem cells (iPSCs) by forced expression of transcription factors (Oct4, Sox2, Klf4, c-Myc) is accompanied by oxidative stress and DNA damage. The DNA repair mechanism of DSBs is therefore critical in determining the genomic stability and efficiency of iPSCs generation. Maintaining genomic stability in PSCs plays a pivotal role in the proliferation and pluripotency of PSCs. In terms of therapeutic application, genomic stability is the key to reducing the risks of cancer development due to abnormal cell replication. Over the years, we and other groups have identified important regulators of DNA damage response in PSCs, including *FOXM1*, *SIRT1* and *PUMA*. They function through transcription regulation of downstream targets (*P53*, CDK1) that are involved in cell cycle regulations. Here, we review the fundamental links between the PSC-specific HR process and DNA damage response, with a focus on the roles of *FOXM1* and *SIRT1* on maintaining genomic integrity.

## 1. Introduction

Embryonic stem cells (ESCs) of mice [1] or humans [2] are derived from the ICM of blastocysts. They hold great promise in cell-based therapy because of their pluripotent nature and the ability to proliferate indefinitely. Since the advent of ESCs decades ago, a massive number of protocols were developed to drive them into different germ layers (endoderm, mesoderm and ectoderm) through manipulation of cell signaling pathways. For instance, we [3] and other research groups [4,5] have reported the differentiation of human ESCs (hESCs) into pancreatic lineages. Extensive work was also done to drive hESCs into trophoblast [6,7] and germ cell [8] lineages. Therefore, ESC is an excellent in-vitro model for understanding early developmental events and their interactions with external stimuli [3,9]. Notwithstanding the potential applications of ESCs in regenerative medicine, tissue rejection in recipients could be a main concern. In 2006, Yamanaka and his team reported the generation of induced pluripotent stem cells (iPSCs) from mouse embryonic and adult fibroblasts through induction of transcription factors (Oct4, Sox2, Klf4 and c-Myc) [10]. A year later, they also reported the generation of iPSCs from human fibroblasts [11]. As patient-specific iPSCs can be derived, these ground-breaking iPSCs solved the major hurdle of tissue rejection in cell-based therapy.

Understanding the biological characteristics of pluripotent stem cells (PSCs) is imperative for exploring their potential applications. In fact, both ESCs and iPSCs can proliferate rapidly. The doubling time of PSCs ranges from 8–10 h, which is much faster than that in somatic cells (around 20 h) [11,12,13]. Interestingly, the fast cell division is reminiscent of early embryonic cells, with doubling time of 5–10 h before implantation [14]. Studies suggested that the rapid proliferation of PSCs was due to their unique cell cycle regulations. For instance, PSCs have a shortened G1 phase as a result of low expression of G1 phase-specific cyclin D and cyclin-dependent kinases (CDK) 4 and 6. The interplay between cyclins, CDK and cyclin-dependent kinase inhibitors (CDKN) is important for tight regulation of cell cycle progression [15,16]. Cell cycle regulation is highly related to pluripotency in PSCs, as the master pluripotent marker *Oct4* controls the progression of cell cycle [17]. In addition, the cell cycle regulators have important functions in DNA damage response (DDR) of PSCs [18]. Replication stress and DNA damage were induced by the shortened G1 phase in mouse ESCs (mESCs) [19]. More importantly, the expression levels of DNA damage marker γH2AX increased during the reprogramming process into iPSCs [20]. Thus, the DDR system in PSCs is essential for minimizing the accumulation of DNA mutations and maintaining genomic integrity in the highly proliferating cells [21]. In this review, we discuss the relationship between cell cycle regulation and DDR in PSCs and the recent discoveries of important regulators, including *FOXM1*, *SIRT1* and *PUMA*, in regulating DDR in PSCs.

## 2. Cell Cycle Regulation in Pluripotent Stem Cells (PSCs)

The mitotic cell division is the most fundamental process for all cell types. It is tightly regulated by the activation and deactivation of cyclin-dependent kinases (CDK) and the oscillatory expression of cyclins at different stages of the cell cycle. The canonical cell cycle in somatic cells consists of a DNA synthesis phase (S phase) and a cell division phase (M phase). G1 (between M and S phase) and G2 (between S and M phase) are two gap phases in between S and M phases. In each cell cycle, cyclin D and its CDK partner CDK4/6 are highly expressed in the G1 phase. Cyclin E and its partner CDK2 are predominantly active between the late G1 and S phase, while cyclin A/E with its partner CDK2 are mainly active between the S and G2 phase. Lastly, cyclin B and its partner CDK1 mainly regulate the G2 and M phase [22]. The oscillatory appearance of cyclins and CDKs are important for ensuring the correct sequences of DNA synthesis prior to cell division, thus controlling genomic integrity. Early studies in mouse blastomeres showed that their cell cycle closely resembled that of the canonical model. Later, it was found that the mouse embryonic cells have rapid cell divisions with doubling time of 5–10 h, owing to the shortened and truncated G1 and G2 phases [23].

### 2.1. Cell Cycle Regulation in mESCs

The fast cell cycle of mESCs is accompanied by a shortened G1 phase. Interestingly, the oscillatory expression and activities of the cyclin–CDK complexes in mESCs are quite different from that in somatic cells [24,25]. First of all, since the G1 phase is significantly shortened, the cyclin D (i.e., cyclin D1 and D3) is expressed at low levels. Throughout the cell cycle, Cdk6 becomes the predominant partner at the G1 phase with an oscillatory expression pattern. On the other hand, cyclin A/E and their partner Cdk2 are expressed at high levels in mESCs. The high expression is cell cycle independent and without oscillation. Lastly, the expression patterns of the mitotic cyclin B and its partner Cdk1 are similar to those in somatic cells; they have oscillatory expression throughout the cell cycle and are only expressed highly in the G2/M phase [24,25].

In addition to the tight regulations of the expression of the cyclin–CDK complexes, another key cell cycle regulator is the retinoblastoma protein (RB). In somatic cells, RB is unphosphorylated and active in the G1 phase. The active RB couples with E2F and binds to the promoters of target genes expressed in the G1/S phase, such as components of the cyclin A/E-Cdk2 complex. The binding of E2F at the promoters leads to histone deacetylation and repression of gene expression. When the cells are ready to enter the S phase, the RB is phosphorylated and does not repress the cyclin A/E–Cdk2 complex when entering the S phase [26]. The hypo-phosphorylation of RB at the G1 phase and the hyper-phosphorylation of RB at the S phase add another level of cell cycle checkpoint to ensure proper cell cycle progression. Interestingly, the inactive hyper-phosphorylated RB is found in mESCs throughout different phases of the cell cycle. The reason is due to the high expression and activities of the cyclin A/E–Cdk2 complex, which lead to phosphorylation of RB [27].

### 2.2. Cell Cycle Regulation in hESCs

Human ESCs, though derived from the ICM of human blastocysts, are considered as being at a primed state similar to that of mouse epiblast stem cells (mEpiSCs). On the other hand, mESCs are generally defined as being in a naïve state [28]. It is therefore reasonable that there are major differences in cell cycle regulation between mESCs and hESCs. Indeed, hESCs have a longer and more functional G1 phase compared with mESCs. Therefore, the cyclin Ds (i.e., cyclin D1, D2 and D3) have intermediate expression in the G1 phase in hESCs, though their expression is still lower than that in somatic cells. Unlike the mESCs, the expression of cyclin D partner CDK4 is higher than CDK6 in hESCs [13,15]. Another major difference between mESCs and hESCs is that the expression pattern of the cyclin A/E–CDK2 complex is cell cycle dependent in hESCs, but it is constant in mESCs. Moreover, the functional RB checkpoint at the G1 phase guarding the correct entry into S phase occurs in hESCs but not in mESCs [15]. On the other hand, there is similarity between mESCs and hESCs; the oscillatory patterns with high expression of the mitotic related cyclin B and its partner CDK1 are observed at the G2/M phase. The expression patterns of cyclin, CDKs and RB in mESCs and hESCs are summarized in Table 1.

### 2.3. Cell Cycle Regulation and Pluripotency

Octamer-binding transcription factor 3/4 (*OCT4*) or POU class 5 homeobox 1 (*POU5F1*) is the most critical transcription factor for maintaining the pluripotency in both mESCs and hESCs [1,2]. Knockout of *OCT4* leads to loss of pluripotency [29,30,31]. In the past decade, research on *OCT4* focused on its role in cell cycle regulation. For instance, *OCT4* downregulates the expression of cyclin D1 for shortening the G1 phase in hESCs. The mediation is through transcriptional regulation of the mir-302 cluster by *OCT4* and depletion of the miR-302 cluster that extends the G1 phase in hESCs [32], highlighting the importance of *OCT4* in cell cycle regulation. In mESCs, on the other hand, *Oct4* activates the expression of *E2f3a*, which positively regulates the transcription of cyclin A and Cdk1 for the fast proliferation of mESCs [33]. It should be noted that other pluripotent markers such as *NANOG* and *SOX2* are also important in cell cycle regulation through interaction with other modulators [34,35]. Collectively, the current evidence shows that pluripotency and cell cycle regulation are highly connected in PSCs.

## 3. DNA Damage Response and Cell Cycle Regulation

### 3.1. Cell Cycle Checkpoints and DNA Damage Response

Apart from the cyclin–CDK complexes and the RB protein, the cyclin-dependent kinase inhibitors (CDKN) are also important for cell cycle progression. Generally, there are two major groups of CDKNs, including the CDK interacting protein/kinase inhibitory protein (CIP/KIP) and the inhibitors of the CDK4/alternate reading frame (INK4/ARF) family. The CIP/KIP family consists of subunits *P21*(*CIP1*), *P27*(*KIP1*) and *P57*(*KIP2*), while the *INK4*/*ARF* family consists of *P16*(*INK4A*), *P15*(*INK4B*), *P18*(*INK4C*) and *P19*(*INK4D*/*ARF*) [25]. CDKNs inhibit the activities of cyclins and CDKs. As the levels of CDKNs in PSCs are low [27], the expression of cyclin–CDK complexes can be maintained at high levels for fast cell cycle progression. A study even suggested that some CDKNs might not be functional in mESCs, as the cyclin D3-Cdk6 expression was not affected by overexpression of its upstream CDKN regulator *p16* [36].

The CDKNs not only act as cell cycle checkpoints but also are heavily linked to DNA damage response. For instance, *P53* is a tumor suppressor gene and responsible for regulating genome stability. In response to DNA double-strand breaks (DSB), *P53* can directly activate the ataxia-telangiectasia mutated (ATM) kinase through phosphorylation. The ATM kinase is then recruited to the site of DNA damage for repairing, either through homologous recombination (HR) or non-homologous end joining (NHEJ) [37]. More importantly, *P53* also activates *P21*, which in turn inhibits cyclin A/E–CDK2 activity, leading to blockage of G1/S phase entry [38]. In PSCs, *P53* is activated during DNA damage. The induction of *P53* in turn suppresses pluripotent marker (*OCT4* and *NANOG*) expression in mESCs and hESCs, leading to their differentiation [39].

### 3.2. DNA Repairing Mechanisms in PSCs

A precise DNA repair system is imperative in PSCs. The system allows the cells to cope with DNA lesions and maintain their genomic integrity during rapid cell cycle progression. In PSCs, the system is tightly controlled by DNA damage response (DDR) signaling. Generally, PSCs respond to DNA damage or lesions through DDR, leading to cell cycle arrest and increased expression of DNA repair genes [40]. Cell cycle arrest at either the G1/S or the G2/M-phase allows incorporation of different DNA repair mechanisms, including mismatch repair (MMR), base excision repair (BER), nucleotide excision repair (NER), HR and NHEJ.

The PSCs have a short G1 phase that can help to minimize the induction of differentiation-related signaling. Therefore, some of the DNA repair mechanisms occurring at the G1 phase checkpoint in somatic cells are bypassed in PSCs. The prolonged S phase makes the PSCs utilize HR preferentially over other DNA repair pathways during DDR [41]. The genes involved in HR, including *RAD51* and *RAD52*, are highly expressed in the S phase of DNA repair. In addition to these genes, the MRE11- RAD50-NBS1 (MRN) complex is also involved in DNA repair through HR and NHEJ. They serve as a DNA damage sensor and generate single stranded DNA regions that activate the checkpoint responses. Following the activation, the checkpoint transmits and amplifies the signal to downstream targets such as the cyclin–CDK complex and other DNA repair-related genes [42,43].

## 4. Critical Regulators of DNA Damage Response in PSCs

### 4.1. FOXM1

Forkhead box (FOX) is a superfamily of transcription factors widely expressed in many tissues. Members of the superfamily share an evolutionary conserved winged-helix DNA-binding domain. To date, more than 50 FOX proteins are identified and are sub-grouped from FOXA to FOXS based on their sequence homologies. They are involved in many cellular processes, including cell cycle progression, angiogenesis, apoptosis and response to DNA damage [44].

Forkhead box M1 (*FOXM1*) is a member of the FOX superfamily consisting of a N-terminal repressor domain and a C-terminal transactivation domain. *Foxm1* is crucial for embryogenesis in mice. *Foxm1* is highly expressed in the epithelial and mesenchymal cells during early embryo development. The patterned expression is important for further development into organs such as liver, lung, intestine and urinary tract [45]. The expression of *Foxm1* in differentiated adult tissue is low, and increases in *FOXM1* expression are correlated with the initiation of cancer formation and tumor initiation [46,47]. We have identified *FOXM1* to be highly expressed in hESCs [48]. In the following sections, we discuss the roles of *FOXM1* in DDR and cell cycle regulation in PSCs.

#### 4.1.1. *FOXM1* and DNA Damage Response in PSCs

*FOXM1* is one of the master regulators in initiating DDR. *Foxm1*-deficient mouse embryonic fibroblasts (MEFs) have more DNA breaks than wildtype MEFs, as shown by increased level of γH2AX. *FOXM1* transcriptionally activates X-ray cross-complementing group 1 (*Xrcc1*) and breast cancer-associated gene 2 (*Brca2*), which are involved in base excision repair and HR repair, respectively, for DSBs [49]. Concerning the HR repair pathway, *FOXM1* upregulates the expression of *NBS1* in human breast cancer cells [50]. *NBS1* is a subunit in the MRN complex responsible for DSB repair through HR and NHEJ. *FOXM1* also enhances the recruitment of the ATM kinase at the sites of DNA damage [50]. The ATM kinase then promotes the phosphorylation of a number of proteins, including P53, H2AX, BRCA1 and NBS1 that are involved in cell cycle arrest and DNA repair [51]. Conversely, depletion of *FOXM1* and *NBS1* through an si-RNA approach led to the loss of HR repair ability in breast cancer cells, resulting in accumulation of γH2AX foci and induction of cellular senescence [50].

**Figure 1:**
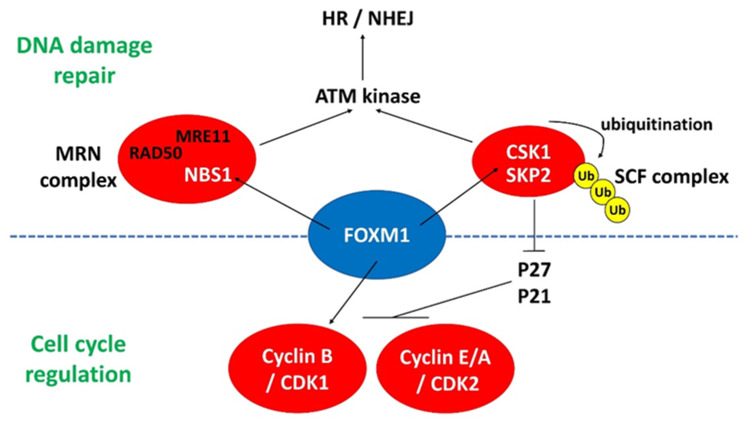
The interactions of *FOXM1* with different regulators involved in DNA damage repair and cell cycle progression. MRN complex: MRE11/RAD50/NBS1; HR: homologous recombination; NHEJ: non-homologous end joining; Ub: ubiquitination.

*FOXM1* also regulates HR repair through transcriptional activation of S-phase kinase-associated protein 2 (*SKP2*) and cyclin-dependent kinases regulatory subunit 1 (*CKS1*). In human cancer cells, *FOXM1* binds to the promoters of *SKP2* and *CKS1* and activates them [52]. Both *SKP2* and *CKS1* are important subunits of the Skp1-Cullin 1-F-box (SCF) ubiquitin ligase complex that interacts with NBS1 and triggers its ubiquitination upon DNA DSB. This process facilitates the recruitment of ATM ligase at the sites of DNA damage and hence induces DNA repair through HR and NHEJ [53]. As most of the studies were performed in human cancer cells, the exact roles of *FOXM1* in DDR in ESCs or during reprogramming to iPSCs are yet to be investigated. However, DNA damage is apparent during acquisition of pluripotency from somatic cells such as MEFs [20]. It is believed that *FOXM1* should play similar roles in regulating DDR in ESCs, as described above. The known interactions of *FOXM1* with DDR-related genes are summarized in Figure 1.

#### 4.1.2. *FOXM1* and CDKs in PSCs

DDR and cell cycle regulation are highly interconnected; therefore, *FOXM1* also interacts with a couple of cyclin-dependent kinases (CDK) (Figure 1). *FOXM1* activates *SKP2* and *CKS1* in human cancer cells. The elevated *SKP2* and *CKS1* downregulates the expression of *P27* and *P21* that is mainly responsible for inhibiting the activity of cyclin E/A–CDK2 and cyclin B–CDK1 complexes. As a result, the activities of the two cyclin complexes are dramatically reduced in *FOXM1*-depleted human cancer cells and MEFs [53]. There is not much research focusing on the roles of *FOXM1* in cell cycle regulation of PSCs. *FOXM1* binds to gene promoters that regulate cell cycle progression, including *CDK12*, *P21*, *CDC20*, and *CDC5* in hESC-derived retinal pigment epithelium (hESC-RPE) cells [54]. Since both CDKs and CDKNs are transcriptionally regulated by *FOXM1*, their combined effects on the enhanced proliferation of hESC-RPE cells remain to be determined. We have reported the roles of *FOXM1* in hESCs [48]. *FOXM1* is highly expressed in undifferentiated hESCs, with a higher expression level in the G2/M phase than the G1 and S phase. Through chromatin immunoprecipitation sequencing (ChIP-seq), we found for the first time that *FOXM1* bound to the promoters of cyclin B1 (CCNB1) and CDK1 in the undifferentiated hESCs, and that depletion of *FOXM1* led to impaired proliferation in hESCs. The study provides direct evidence that *FOXM1* regulates cell cycle progression through transcription activation of the cyclin–CDK complex. The interactions of *FOXM1* with various cell cycle regulators are shown in Figure 1.

### 4.2. SIRT1

Sirtuin 1 (*SIRT1*) belongs to the sirtuin family. The human sirtuin family consists of seven members (*SIRT1* to *SIRT7*). They have highly conserved domain that acts as a nicotinamide adenosine dinucleotide (NAD)-dependent histone deacetylase [55]. Among the sirtuin members, *SIRT1* is the most widely studied molecule. It is detected in the nucleus and involved in many cellular events including transcriptional silencing, DNA damage repair, cell cycle regulation, insulin regulation and longevity [56,57,58,59,60]. As a histone deacetylase, *SIRT1* is recruited to the chromatin and deacetylates histone 1 lysine 26 (H1K26), H3K9, H3K14 and H4K16 [61]. In addition, *SIRT1* also deacetylates a number of transcription factors, including P53, FOXO, p300 histone acetyltransferase and E2F transcription factor 1 (E2F1) [62]. We and others have demonstrated that *SIRT1* is highly expressed in both mESCs and hESCs [56,57,63]. In the following section, we review the importance of *SIRT1* in regulating cell cycle progression and DDR.

#### 4.2.1. *SIRT1* and *P53*

*P53* is important for cell cycle regulation and DDR. *SIRT1* is an important regulator of *P53* in PSCs and highly expressed in undifferentiated hESCs. It inactivates *P53* expression through its protein deacetylase property. *OCT4* regulates the activity of *SIRT1*. Downregulation of *OCT4* reduces *SIRT1* expression and upregulates P53 activity. Consistently, depletion of *SIRT1* in hESCs led to stabilization of the P53 protein and hence its activity due to acetylation at the K120 and K164 sites [64]. It is noted that *SIRT1* is important for cell survival in undifferentiated but not in differentiated cells. The depletion of *SIRT1* upregulates P53 activity in the undifferentiated hESCs but not in other cell types, leading to programmed cell death related to decreased expression of DNA repair enzymes such as *MSH2*, *MSH6* and *APEX1* in hESCs [65].

*SIRT1* is also the upstream regulator of p53 in miPSCs [57]. A similar relationship between *SIRT1* and p53 is found during reprogramming of mouse MEFs into iPSCs. The expression of *SIRT1* level is increased during reprogramming of mouse MEFs. The deacetylation of p53 by *SIRT1* increases the expression of Nanog, which facilitates the reprogramming of MEF. On the other hand, inhibition of *SIRT1* by miR-34a reduces the reprogramming efficiency [57]. 

#### 4.2.2. *SIRT1* and DNA Damage Response Genes

Given the importance of *SIRT1* in efficient reprogramming of MEFs into miPSCs and increased DNA damage markers such as γH2AX during the iPSC reprogramming process [20], *SIRT1* should have some roles in DDR during reprogramming. In fact, *SIRT1* is responsible for deacetylation of NBS1 in the MRN complex, leading to phosphorylation of NBS1 for recruiting ATM kinase to the site of DNA damage [66]. Either NBS1 depletion or its hyperacetylation reduces DNA repair and cell survival [66]. In addition, *SIRT1* also deacetylates another important DNA repair partner WRN protein and promotes its translocation for DNA repair [67,68].

**Figure 2:**
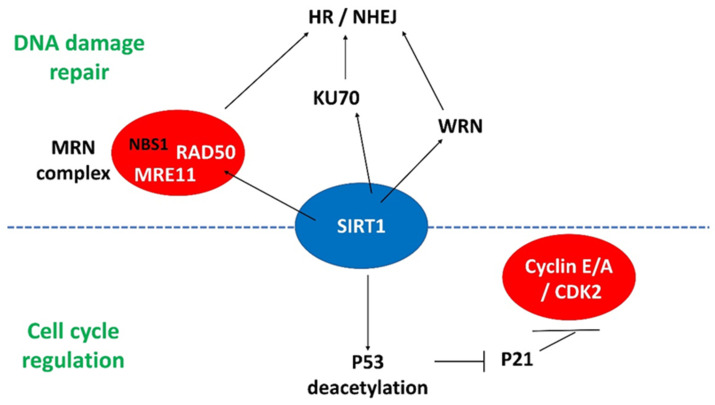
The interactions of *SIRT1* with different regulators involved in DNA damage repair and cell cycle progression. MRN complex: MRE11/RAD50/NBS1; HR: homologous recombination; NHEJ: non-homologous end joining.

Recently, we found that miR-135a regulated *SIRT1* expression during miPSCs reprogramming. By immunoprecipitation, *SIRT1* was found to interact with Wrn and Ku70 to form a protein complex in the initial phase of reprogramming [56]. The Wrn protein interacts with the Ku70/80 heterodimer [69], indicating that Wrn has a direct role in NHEJ. Thus, our results suggested direct involvement of *SIRT1* in DSB repair during reprogramming. The increase in DNA repair in turn improved the reprogramming efficiency. Apart from Ku70 and Wrn, the MRN complex is important in HR-mediated DSB repair [70]. We found that the MRN complex components Mre11 and Rad50 were included within the complex of *SIRT1* during reprogramming. Added together, the findings suggested that *SIRT1* is an important regulator interacting with different complexes for DNA repair in response to DNA damage. The interactions of *SIRT1* with various cell cycle and DNA damage response regulators are illustrated in Figure 2.

### 4.3. Interplay between FOMX1 and SIRT1

Though *FOXM1* and *SIRT1* regulate different downstream modulators during cell cycle progression and DDR, these two modulators might be connected. In human breast cancer tissues, *FOXM1* protein expression level is highly correlated with that of *SIRT1* [71]. It was reported that *SIRT1* deacetylated and reduced expression of FOXO3. As FOXO3 negatively regulates *FOXM1*, the reduced FOXO3 expression level in turn increased *FOXM1* level, which suggested *SIRT1* might indirectly induce *FOXM1* expression [72]. However, opposite findings that *SIRT1* reduced *FOXM1* expression were also reported. In breast cancer cells, *SIRT1* was found to deacetylase mitogen-activated protein kinase 1 (MAP2K1), leading to inactivation of the MEK/ERK cell signaling pathway and reduction in *FOXM1* protein. Consistently, the phenomenon was also observed in MEFs, indicating that the relationship was not restricted to cancer cells [73]. Another study even showed that *SIRT1* directly bound to and deacetylated *FOXM1* to suppress its expression [74].

*FOXM1* was also reported to be the upstream regulator of *SIRT1*; *FOXM1* regulated the chromatin structure remodeling and induced transcriptional activation of *SIRT1* [75]. In cancer glioma cells, downregulation of *FOXM1* by siRNA reduces *SIRT1* expression. Critically, a *FOXM1* binding site on the *SIRT1* promoter has been identified [75]. The relationship between *FOXM1* and *SIRT1* in PSCs remains largely unknown. More mechanistic studies are required to investigate whether a feedback loop exists between the two genes and how their interplay affects DDR in PSCs.

### 4.4. PUMA

P53 upregulated modulator of apoptosis (*PUMA*) is another factor that recently emerged to regulate cell cycle progression and inhibit DDR in PSCs. As the name implies, *PUMA* is a direct target of P53. It has been implicated in causing cell apoptosis following irradiation in intestinal progenitor cells [76] and hematopoietic stem cells [77]. *PUMA* knockout in human iPSCs enhances DNA repair abilities of the cells, as shown by decreased γH2AX-positive cells when compared with the wildtype cells after irradiation. Mechanistic analysis demonstrated that *PUMA* formed a protein complex with early mitotic inhibitor 1 (EMI1) and RAD51 in the cytoplasm of PSCs and promoted the ubiquitination and degradation of RAD51. The relationship was supported by an increase in RAD51 nuclear translocation post-irradiation in the *PUMA*^–/–^ PSCs [78]. Since RAD51 is a DDR-related protein responsible for repairing through HR [79], the increased cell survival following irradiation in the *PUMA*-deficient cells could be the result of enhanced DNA repair. We have previously reported that P53 was a target of *SIRT1* [57]. Coincidently, *SIRT1* inhibition upregulates *PUMA* through modulating P53 activity [65]. It is therefore possible that *SIRT1* could be the upstream regulator of *PUMA* in PSCs for mediating DDR.

## 5. Concluding Remarks

In this article, we reviewed the unique features of cell cycle regulation and DDR in PSCs. The observations showed that the regulatory mechanisms are tightly related to the maintenance of pluripotency. We also summarized research on how *FOXM1*, *SIRT1* and *PUMA* regulated cell cycle and DDR in PSCs. Further research on the mechanisms in preserving genome integrity of PSCs could possibly provide new insights into PSCs application in cell-based therapy and a better understanding of the longevity of cells.

## Figures and Tables

**Table 1:** Expression levels of cyclins, cyclin-dependent kinases (CDKs) and phosphorylation status of retinoblastoma protein (RB) in mESCs and hESCs during cell cycle progression.

	mESCs	hESCs
Cyclin expression levels		
Cyclin A/E	High, non-oscillatory	High, oscillatory
Cyclin B	High, oscillatory	High, oscillatory
Cyclin D	Low, oscillatory	Intermediate, oscillatory
CDKs expression levels		
CDK1	High, oscillatory	High, oscillatory
CDK2	High, non-oscillatory	High, oscillatory
CDK4	Low, oscillatory	Medium, oscillatory
CDK6	Medium, oscillatory	Low, oscillatory
RB phosphorylation		
RB	Hyper-phosphorylated	Hypo-/Hyper-phosphorylated

## Data Availability

Not applicable.
